# Multiyear data on benthic foraminifera in a glaciated fjord of Svalbard

**DOI:** 10.1016/j.dib.2018.01.046

**Published:** 2018-02-03

**Authors:** Elena Golikova, Olga Kniazeva, Sergei Korsun

**Affiliations:** a P.P. Shirshov Institute of Oceanology of Russian Academy of Sciences, Russia; b Saint-Petersburg State University, Russia

## Abstract

Glaciers in the fjords of Svalbard have been receding over last decades. Tempelfjorden, a typical glaciated fjord in West Spitsbergen (78°24′06″ N, 17°02′30″ E), has been sampled in summer 1995 and 2001–2007 for modern benthic foraminifera. We have normalized the abundances and unified the taxonomy of all these published and unpublished data sets and then compiled the record of foraminiferal assemblages changing over years into a comprehensive database. The record includes data on living and dead abundances of benthic foraminiferal species in the surface sediments (0–2 cm) and downcore abundances of living foraminifera (only for 2004). This database portrays benthic foraminifera, this key group of microfossils, in a gradually changing Arctic environment.

**Specifications Table**

**Table:** 

Subject area	*Earth and Planetary Sciences*
More specific subject area	*Benthic Foraminifera*
Type of data	*Tables, figure*
How data was acquired	*Sampled in summer 1995 and 2001–2007 using box or interface corers; samples stained with Rose Bengal; live and dead benthic foraminifera identified to the species level and counted.*
Data format	*Tables with densities of live and dead foraminifera; one table listing the dates, locations and water depths of sampling stations.*
Experimental factors	–
Experimental features	–
Data source location	*Tempelfjorden, the Svalbard archipelago*
Data accessibility	*The data are available with this article*

**Value of the data**

• The data make it possible to link the multiyear dynamics of benthic foraminiferal assemblages and glacier retreat.
• The data allow assessing the response of foraminiferal assemblages to surge events of the glacier in the fjord head.
• The data on vertical distribution of live benthic foraminifera in 10 cm cores can be used to reveal species-specific microhabitat preferences in a glaciated environment.

## 1 Data

Fjords are natural archives of sediments that can provide high-resolution records of late- and postglacial palaeoceanographic changes. Today, subpolar fjords are often affected by glacial sedimentation [1] with glaciers delivering substantial amount of mineral matter. Turbid meltwater is the main sources of environmental stress for the benthic organisms. This stress affects the structure of modern benthic assemblages [2], [3], [4], [5]. Studies of present-day processes in subpolar glaciated fjords are essential for accurate interpretation of past environmental records [6].

Two glaciers terminate in the head of Tempelfjorden, the tidewater Tunabreen merged with the land-based Von Postbreen (see [4] and [8] for the detail). Both glaciers have experienced several surges over the last two centuries [7]. Previous surveys recorded substantial changes in benthic foraminiferal assemblages along the Tempelfjorden in 1995 and 2006 [4], [8]. The data presented here cover the modern surface and downcore distribution of living and dead benthic foraminifera in Tempelfjorden in years 1995 and 2001–2007. The species list is taxonomically verified, and all abundances are normalized to 10 cm^3^.

The dataset consists of:

1. A station list showing sampling dates, locations and water depths (Table 1),

**Table 1:** Station list.

**1995**					**2001**				
St.	Latitude N	Longitude E	Water depth [m]	Date	St.	Latitude N	Longitude E	Water depth [m]	Date
					**757**	78°26.26′	17°22.95′	26	8/31/2001
**53**	78°26.25′	17°21.49′	46	8/23/1995	**758**	78°26.25′	17°20.74′	46	8/31/2001
**54**	78°26.00′	17°17.00′	40	8/23/1995	**759**	78°26.01′	17°16.98′	34	8/31/2001
**55**	78°25.42′	17°13.00′	37	8/23/1995	**760**	78°25.38′	17°12.45′	29	8/31/2001
**57**	78°25.05′	17°08.26′	67	8/23/1995	**761**	78°25.05′	17°08.36′	71	8/31/2001
**58**	78°23.53′	16°58.44′	99	8/23/1995	**762**	78°23.49′	16°58.06′	104	8/31/2001
**59**	78°22.05′	16°51.08′	103	8/23/1995	**763**	78°21.58′	16°49.55′	80	8/31/2001
**60**	78°22.01′	16°40.26′	97	8/23/1995	**764**	78°22.14′	16°40.23′	92	8/31/2001
**61**	78°21.57′	16°28.12′	50	8/23/1995	**765**	78°21.58′	16°27.54′	65	8/31/2001
**2002**					**2003**				
St.	Latitude N	Longitude E	Water depth [m]	Date	St.	Latitude N	Longitude E	Water depth [m]	Date

					**61**	78°26.26′	17°22.84′	25	7/22/2003
					**60**	78°26.26′	17°22.72′	31	7/22/2003
					**59**	78°26.02′	17°16.89′	40	7/22/2003
					**58**	78°25.37′	17°12.90′	26	7/22/2003
**19**	78°24.89′	17°08.21′	72	7/6/2002	**57**	78°25.05′	17°08.00′	73	7/22/2003
**18**	78°24.48′	16°58.98′	92	7/6/2002	**55**	78°23.50′	16°58.82′	94	7/22/2003
**17**	78°22.19′	16°54.08′	97	7/6/2002	**54**	78°21.95′	16°50.91′	101	7/22/2003
**16**	78°21.99′	16°40.61′	49	7/6/2002	**53**	78°22.12′	16°49.28′	99	7/22/2003
					**52**	78°21.54′	16°28.17′	63	7/22/2003
**2004**					**2005**				
St.	Latitude N	Longitude E	Water depth [m]	Date	St.	Latitude N	Longitude E	Water depth [m]	Date

					**013**	78°26.23′	17°19.51′	n/a	7/8/2005
**242**	78°25.98′	17°09.00′	37	7/9/2004	**012**	78°25.96′	17°17.11′	n/a	7/8/2005
**241**	78°25.52′	17°12.63′	30	7/9/2004	**011**	78°25.42′	17°12.96′	n/a	7/8/2005
**240**	78°25.14′	17°07.88′	71	7/9/2004	**010**	78°25.04′	17°08.51′	n/a	7/8/2005
**239**	78°23.63′	16°57.86′	101	7/9/2004	**009**	78°23.57′	16°58.52′	n/a	7/8/2005
**238**	78°21.87′	16°51.13′	100	7/9/2004	**008**	78°22.01′	16°51.39′	n/a	7/8/2005
**237**	78°22.05′	16°39.72′	87	7/8/2004	**007**	78°22.05′	16°40.72′	n/a	7/8/2005
**236**	78°21.63′	16°27.68′	65	7/8/2004	**006**	78°21.58	16°28.27′	n/a	7/8/2005
	
**2006**					**2007**				
St.	Latitude N	Longitude E	Water depth [m]	Date	St.	Latitude N	Longitude E	Water depth [m]	Date

**188**	78°26.45′	17°18.97′	40	8/4/2006	**8**	78°26.24′	17°19.16′	45	7/3/2007
**186**	78°25.96′	17°17.27′	36	8/4/2006	**7**	78°26.00′	17°17.00′	40	7/3/2007
**190**	78°25.51′	17°13.81′	38	8/4/2006	**6**	78°25.42′	17°13.00′	37	7/2/2007
**184**	78°24.96′	17°08.51′	71	8/4/2006	**5**	78°25.05′	17°08.26′	67	7/2/2007
**182**	78°23.34′	16°58.97′	88	8/4/2006	**4**	78°23.53′	16°58.44′	99	7/2/2007
**180**	78°22.02′	16°50.94′	101	8/4/2006	**3**	78°22.05′	16°51.08′	103	7/2/2007
**178**	78°22.11′	16°40.56′	95	8/4/2006	**2**	78°22.10′	16°40.26′	97	7/2/2007
**191**	78°21.68′	16°28.15′	57	8/4/2006	**1**	78°21.57′	16°28.12′	50	7/2/2007
2. Abundances of living and dead foraminifera (size fraction > 0.125 mm) from surface samples along the Tempelfjorden obtained in summer 1995 and 2001–2007 including numbers of counted specimens per sample, percentage of calcareous species and percentage of the specimens > 1 mm (Table 2, Table 3, Table 4, Table 5, Table 6, Table 7, Table 8, Table 9, Table 10),

**Table 2:** Densities of benthic foraminifera from sediment-surface samples retrieved in 1995 (living only), 2001 (living only) and 2002 (living and dead).

**Year**	**1995**								**2001**								**2002**							
	**Live foraminifera (N)**								**Live foraminifera (N)**								**Live foraminifera (N)**				**Dead foraminifera (N)**			
**Station no.**	61	60	59	58	57	55	54	53	765	764	762	761	760	759	758	757	16	17	18	19	16	17	18	19
*Adercotryma glomerata*	1.6	2.8							3.7	1.7	2.0							0.4	0.1			0.6	0.2	
*Ammodiscus catinus*										1.7	0.7	0.7								0.1				
*Ammodiscus sp.*	0.6	4.8		2.5														0.2	0.1			0.8	0.2	
*Ammotium sp.*									6.2												0.4			
*Angulogerina fluens*	3.9								2.5															
*Armorella sp.*																							0.2	
*Astacolus hyalacrulus*											0.7													
*Astrononion gallowayi*	2.6		0.5	0.6					4.9									0.2			0.8	0.8	0.2	
*Buccella frigida*	0.6	9.0	4.5	2.5	1.8				1.2	5.2	14.0	24.3	0.3		0.2			0.4	0.6	2.6	1.6	5.4	3.4	2.2
*Buccella tenerrima*	0.6								1.2															
*Cassidulina reniforme*	1.0	22.8	34.0	308.8	22.8	18.4	10.5		13.5	13.9	11.3	138.6	71.0	11.4	44.3	11.1	1.6	0.2	0.3	14.2	16.8	32.6	26.6	19.1
*Cibicides lobatulus*	0.3																				0.4			
*Cornuspira foliacea*													0.3					0.2	0.1	0.3		0.2		
*Cornuspira sp.*									1.2		0.7									0.1				
*Cuneata arctica*	0.6	0.7																						
*Cyclogyra involvens*		0.7	1.5	0.6																				
*Dentalina frobisherensis*		1.4																						
*Dentalina ittai*				0.6																				
*Dentalina pauperata*				1.3	0.3	0.7																		
*Dentalina spp.*									1.2	1.7				0.1										
*Elphidiidae sp.*																							0.2	
*Elphidium bartletti*	0.3	26.9	1.0	1.9	0.3				2.5		2.0	1.4	0.3						0.7	0.1		0.2	0.2	0.1
*Elphidium excavatum f. clavata*	1.3	23.5	40.5	17.6	53.3	34.6	1.9	19.7	56.6	57.4	50.7	32.9	17.7	26.0	22.2	9.8	8.0	3.4	1.1	7.8	26.8	11.6	5.4	2.5
*Elphidium incertum*											5.3	1.4	1.7					0.8	0.3	2.4		0.2	0.2	0.5
*Elphidium subarcticum*	0.3																							
*Epistominella sp.*	0.3	3.5			0.3																			
*Fissurina laevigata*				3.8																				
*Fissurina marginata*	0.3																							
*Fissurina spp*									1.2		0.7													
*Glandulina laevigata*	1.0	0.7																						
*Globobulimina auriculata*											1.3													
*Globobulimina turgida*																	2.0	1.6	1.4			0.4		
*Guttulina sp.*	2.3	6.2	6.5	2.5	1.6	0.7	0.2																	
*Haynesina orbiculare*												0.7	5.7	0.1										0.1
*Hippocrepina indivisa*	1.9								14.8															
*Hyperammina subnodosa*	0.3	1.4							9.5	2.8														
*Islandiella islandica*																							0.2	
*Islandiella norcrossi f. helenae*	3.2	53.8	28.0	28.4	3.9													3.0	1.9	2.0	2.4	6.6	4.2	2.8
*Islandiella norcrossi f. norcrossi*	2.3																			0.3		1.0	1.0	0.4
*Islandiella norcrossi s.l.*									7.4	47.0	15.3	7.9	1.0	0.4	0.1	0.1				0.1				
*Labrospira crassimargo*	12.0	100.8	39.0	73.7	0.5				123.1	352.2	93.3	3.6	2.3	0.9	0.2	0.4	3.6	12.6	3.0	4.0	13.6	5.2	6.0	1.6
*Labrospira jeffreisi*	1.3	1.4																						
*Lagena distoma*	0.3																							
*Lagena gracillima*				0.6				0.4																
*Lagena laevis*	0.3																							
*Lagena semilineata*				1.3																				
*Lagena spp.*	0.3								1.2			0.7					0.4			0.1				0.2
*Lagena sulcata*	0.3																							
*Melonis barleeanus*																								0.1
*Miliolidae spp.*	0.6																							
*Miliolinella sp. 1*								0.4									0.4	0.8	0.1			0.4		0.2
*Miliolinella sp. 2*																		0.4					0.2	
*Miliolinella subrotunda*		0.7	1.0	6.9	0.5								0.3	0.1						0.1				0.1
*Nodulina? sp.*				0.6																				
*Nonionellina labradorica*	16.5	24.8	44.0	0.6	2.6				4.9	12.2	15.3	17.9	1.3				63.2	13.0	8.1	4.3	34.4	2.0	2.0	1.6
*Polymorphinidae spp.*									1.2	7.0	0.7		1.3	0.2							1.2		0.2	0.1
*Proteonina atlantica*	3.9	0.7																						
*Proteonina sp.*	1.9	5.5		0.6																				
*Psammosphaera sp.*																						0.4	0.4	
*Pyrgo williamsoni*	2.9	7.6	19.5	33.4	7.8	12.7			8.6		8.0	5.7	17.0				1.6	1.2	0.8	1.7	0.4		0.4	0.2
*Quinqueloculina "elongata"*									1.2		0.7	0.7	2.7							0.4				
*Quinqueloculina elongata?*	1.0	5.5	7.0	0.6	2.3																			
*Quinqueloculina seminula*	0.3		1.0	1.9	0.5	0.7			8.6		0.7		4.3					0.4		0.4				
*Quinqueloculina sp.*									1.2	1.7	5.3							0.4			0.4			
*Quinqueloculina stalkeri*	10.3	8.3	13.0	3.8			7.6	20.5	1.2					0.7	0.3	1.3								0.3
*Recurvoides sp.*	1.3	20.0	3.5						2.5	5.2														
*Recurvoides turbinatus*									13.5	36.5	28.7			0.1			3.6	3.4	1.9		1.0	8.6	6.4	
*Reophax cf. fusiformis*																	2.0				4.8	0.4	0.4	
*Reophax dentaliniformis*									2.5													0.6		
*Reophax scorpiurus* s.l.	42.4	3.5		5.0	1.0				30.8	19.1	0.7	0.7							1.1		0.8	0.2		0.1
*Reophax sp.1*	1.6	46.2	16.5																					
*Rhabdammina abyssorum*	11.6								0.5															
*Robertina arctica*	6.8	25.5	9.5						3.7	12.2	0.7									0.1				
*Rosalina sp.*																						0.2		
*Silicosigmoilina groenlandica*									9.8	7.0							1.6			0.1	9.2	0.8		
*Siphonaperta agglutinata*									4.9								0.8				0.8			
*Spiroplectammina biformis*			1.0						2.5				0.3				0.8							
*Stainforthia feylingi*									2.5			0.7	0.3											
*Stainforthia loeblichi*	1.0	11.7	0.5	8.8					1.2									0.2				0.8	1.0	
*Textularia earlandi*	0.3	0.7	3.0	3.2	0.3	2.1		2.0									1.6			0.3	5.2	0.2		
*Triloculina trihedra*	0.3	0.7	4.5	1.3	0.8					3.5										1.6	1.2	0.6		0.1
*Trochammina nana*	1.3								2.5															
*Trochamminella atlantica*									2.5												0.4			
calcareous indefinite									2.5															
**Benthics/10 cm^3^**	144.2	421.7	279.4	513.5	100.4	70.0	20.2	43.1	360.8	588.0	259.3	237.9	128.0	40.1	67.2	22.7	91.2	42.8	21.6	43.1	131.6	80.8	59.2	32.3
**Benthics counted**	459	612	666	817	389	99	117	457	416	369	389	333	384	341	1210	398	228	214	216	431	329	405	296	323
**No. of species**	43	30	22	27	17	7	4	5	38	18	22	15	17	10	6	5	14	19	16	22	20	25	22	19
**% calcareous**	43	55	77	83	98	97	100	95	38	28	52	98	98	97	100	98	86	61	71	90	66	78	77	95
**% > 1 mm**																		0				0.2

**Table 3:** Densities of living and dead benthic foraminifera from sediment-surface samples retrieved in 2003.

	**Live foraminifera (N)**									**Dead foraminifera (N)**								
Station no.	61	60	59	58	57	55	54	53	52	61	60	59	58	57	55	54	53	52
*Adercotryma glomerata*	7.6			1.5						9.8								
*Ammodiscus sp.*	2.2			2.8						2.2			0.5					
*Ammotium cassis*	15.2																	
*Angulogerina fluens*	7.6									10.8								
*Astacolus hyalacrulus*				0.3														
*Astrammina sp.*					0.1					1.1			0.8	0.7				
*Astrononion gallowayi*	2.2			0.3						7.6			0.3	0.1				0.0
*Buccella frigida*		3.4		4.4	5.6	0.1							2.8	1.7				0.0
*Buccella tenerrima*										2.2								
*Cassidulina reniforme*	1.1	42.3	19.9	22.7	38.0	2.2	1.5	9.9	3.1	41.2	4.6	3.9	9.0	35.2	8.0	13.9	1.0	1.6
*Cibicides lobatulus*	4.3		0.6							3.3				0.3				0.0
*Cornuspira foliacea*	0.0		0.0							0.0								
*Cornuspira sp.*				0.3	0.1	0.1				2.2			0.3					
*Dentalina baggi*		0.0		0.0														
*Dentalina frobisherensis*		1.1													0.1			
*Dentalina ittai*					0.1													
*Dentalina pauperata*					0.1	0.1												
*Dentalina spp.*				0.3														
*Egerella advena*	1.1																	
*Elphidium bartletti*		2.3	7.2	3.4	0.1					5.4			0.3					
*Elphidium excavatum*								0.0										0.0
*Elphidium excavatum f. clavata*	16.3	8.0	12.1	4.1	21.4	11.6	0.2	0.6	0.8	190.9	12.6	13.8	11.6	5.9	3.0	1.3	0.2	0.5
*Elphidium incertum*				2.3	1.5	0.1							0.3	0.1				
*Elphidium subarcticum*										2.2	1.1							
*Epistominella vitrea*	1.1									1.1								
*Fissurina spp.*										2.2								
*Glandulina laevigata*														0.1				
*Globobulimina spp.*	2.2	3.4	0.6							2.2								
*Glomospira sp.*										1.1								
*Haynesina orbiculare*			0.6	0.5	0.1	0.7									0.1			
*Hippocrepina indivisa*	1.1									3.3								
*Hyperammina subnodosa*	6.1									3.8								
*Islandiella norcrossi f. helenae*	10.8	42.3	25.9	15.7	8.1	0.5				16.3	2.3		3.6	1.8	0.2			
*Islandiella norcrossi f. norcrossi*	2.2	1.1	0.6	1.5	0.1					8.7		0.6	1.8	0.2	0.1			
*Islandiella norcrossi s.l.*			3.9	1.5	0.1					1.1		0.6	1.3	0.1			0.0	
*Labrospira crassimargo*	36.9	90.4	119.2	113.5	6.1	0.1				53.5	0.1	1.7	12.2	1.5				
*Labrospira jeffreisi*				0.3						2.2								
*Lagena spp.*				0.3	0.1					1.1								
*Miliolidae spp.*						0.1								0.1				
*Miliolinella sp. 1*				0.5	0.1								0.3	0.1				
*Miliolinella sp. 2*		4.6	2.8		2.2			0.1	0.0							1.4		0.1
*Nonionella atlantica*										2.2								
*Nonionella turgida digitata*										2.2								
*Nonionellina labradorica*	9.8	19.4	27.6	3.6	0.8	0.2				53.2		1.7	1.8	1.9	0.1	0.1	0.1	0.3
*Polymorphinidae spp.*	1.1	2.3			0.1		0.1							0.1				
*Psammosphaera sp.*													0.3					
*Pyrgo williamsoni*	2.2	3.5	6.1	11.1	3.9	0.3				1.1			0.8	1.0	0.2			
*Quinqueloculina seminula*	2.2	1.1		0.5	1.2	3.2								0.1	0.8			
*Quinqueloculina sp.*		2.3		0.5	0.3									0.1				
*Quinqueloculina stalkeri*	2.2	5.7	1.7		0.1	0.1	0.4	2.1	1.3	5.4				0.1	1.0	17.3	0.2	0.1
*Recurvoides turbinatus*	36.9	73.1	23.2	41.3		0.1				29.3		0.6	4.6					
*Reophax atlantica*	7.6			0.3						4.3			0.3					
*Reophax dentaliniformis*	1.1			0.3						6.5			0.5	0.1				
*Reophax scorpiurus* s.l.	41.3	4.6	2.8	4.6	0.2					61.8		2.8	3.1	0.3				
*Robertina arctica*	15.2	3.4	5.0															
*Saccamina sp. ('silver Saccamina')*	5.4				0.2	0.1								0.1				
*Saccamminidae spp.*				0.3						1.1								
*Silicosigmoilina groenlandica*	10.8	5.7	3.9	1.0	0.5					15.2		0.6						
*Siphonaperta agglutinata*	9.8									1.1								
*Spiroplectammina biformis*														0.1				
*Stainforthia loeblichi*	2.2			2.1						5.4			1.0				0.0	
*Textularia earlandi*			0.6							5.4				0.2		0.2		0.0
*Triloculina trihedra*			1.1	0.3	0.8					2.2		0.6	0.3	1.2				
*Trochammina bullata*	1.1									1.1								
*Trochammina nana*	9.8			0.3						4.3			0.3					
*Trochamminella atlantica*	3.3									6.5								
agglutinated indefinite				0.5	1.7								0.3					
calcareous indefinite				2.1	0.1		0.1											
**Benthics/10 cm^3^**	279.6	320.2	264.9	244.9	93.7	19.5	2.3	12.7	5.3	583.4	20.7	26.6	58.1	52.7	13.5	34.2	1.4	2.7
**Benthics counted**	437	285	483	950	1453	321	34	407	170	657	21	50	227	817	222	513	46	85
**No. of species**	34	21	21	35	29	16	5	5	4	43	5	10	25	26	10	6	6	10
**% calcareous**	33	46	44	32	91	98	100	100	100	64	99	79	61	94	100	99	100	99
**% > 1 mm**	2.2	0.1	0.0	0.0						0.7	0.5	0.3	0.1

**Table 4:** Densities of living and dead benthic foraminifera from sediment-surface samples retrieved in 2004 (replication A).

	**Live foraminifera (N)**							**Dead foraminifera (N)**						
Station no.	242	241	240	239	238	237	236	242	241	240	239	238	237	236
*Adercotryma glomerata*				0.6		5.7	5.5							6.2
*Ammodiscus sp.*			0.1	1.4	0.5	2.3								
*Ammotium cassis*						12.6	0.6						1.1	
*Angulogerina fluens*						2.3	3.4							9.8
*Astacolus hyalacrulus*														1.2
*Astrononion gallowayi*	0.0						1.2	0.1	0.1		0.6			12.3
*Aubignina sp.*									0.1					
*Buccella frigida*			2.7	1.4			1.8	0.0		0.3	0.9			9.8
*Buccella tenerrima*			0.1											2.5
*Cassidulina reniforme*	3.7	3.9	28.0	10.0	9.1	9.1	2.2	3.4	6.0	16.9	4.0	4.8	18.3	18.5
*Cibicides lobatulus*							0.6	0.0		0.1			1.1	4.9
*Cornuspira sp.*		0.1	0.1				0.3							
*Dentalina baggi*	0.1													
*Dentalina pauperata*	0.1				0.5		0.8							
*Dentalina spp.*		0.1												
*Elphidiidae sp.*														1.2
*Elphidium bartletti*		0.1	0.4	0.6		9.1	0.6	0.0						7.4
*Elphidium excavatum f. clavata*	1.6	8.7	8.1	8.6	1.6	43.4	8.9	0.6	2.5	1.9	2.0		19.4	43.1
*Elphidium incertum*		1.6	3.3	1.4				0.1	0.2	0.6				
*Elphidium subarcticum*							1.2							2.5
*Eponides sp.*											0.3			
*Glandulina laevigata*														1.2
*Globobulimina spp.*				0.9	1.1	2.3	0.9				0.9	0.5	1.1	2.5
*Haynesina orbiculare*		1.3							0.3					
*Hyperammina subnodosa*						1.0	2.8						0.7	2.5
*Islandiella norcrossi f. helenae*		0.7	4.3	9.1	4.8	19.4	4.6			0.9	2.0		4.6	19.7
*Islandiella norcrossi f. norcrossi*			0.6	1.1	1.1	6.9	0.9	0.0	0.1	0.1	0.3		3.4	4.9
*Islandiella norcrossi s.l.*				2.0		5.7	0.6				0.3		1.1	
*Labrospira crassimargo*		0.1		11.1	5.9	91.4	11.7		0.1		6.0	4.8	16.0	32.3
*Labrospira jeffreisi*							0.3							
*Lagena spp.*	0.0						1.2	0.0						2.5
*Miliolidae spp.*							0.9							
*Miliolinella sp. 1*		0.2					0.6		0.2					
*Miliolinella sp. 2*		0.1	0.4	1.1			0.3							
*Nonionella turgida digitata*					0.5	1.1								
*Nonionellina labradorica*		0.2	0.3	11.4	99.2	3.4	39.1	0.5	0.2	0.4	1.4	4.3	12.6	86.2
*Polymorphinidae spp.*		0.3		0.3	0.5	1.1	0.9		0.1					2.5
*Pyrgo williamsoni*	0.0	0.2	1.4	5.4	1.1	5.7	1.5		0.1	0.3				
*Quinqueloculina arctica*			0.1											
*Quinqueloculina seminula*		1.4	1.1		3.2		0.9				0.6	0.5		
*Quinqueloculina sp.*					2.1	1.1	0.6			0.1		0.5	2.3	
*Quinqueloculina stalkeri*	0.3		0.1		0.5			0.3	0.1	0.4		0.5		1.2
*Recurvoides turbinatus*				18.0	2.1	88.0	36.0				0.3		35.4	17.2
*Reophax atlantica*						5.7	1.8						3.4	
*Reophax scorpiurus* s.l.						3.4	4.6						2.3	43.1
*Robertina arctica*						17.1	6.2							
*Saccamina sp. ('silver Saccamina')*			0.1											
*Saccamminidae spp.*		0.1	0.1											
*Silicosigmoilina groenlandica*		0.2	0.7	0.3	0.5	11.4	6.2			0.3		0.5	13.7	7.4
*Siphonaperta agglutinata*							0.6							1.2
*Stainforthia loeblichi*				0.9				0.1			1.1		1.1	4.9
*Triloculina trihedra*		0.1	0.7				0.6	0.0	0.1	0.1	0.3			
*Trochammina nana*				1.4			2.8							3.7
*Trochamminella atlantica*							1.2							
agglutinated indefinite				1.4										
calcareous indefinite	0.0													
**Benthics/10 cm^3^**	5.861	19.33	53.14	88.57	134.4	349.6	155.2	5.167	10.15	22.43	20.86	16.53	137.9	352.3
**Benthics counted**	211	261	372	310	252	312	514	186	137	157	73	31	125	302
**No. of species**	9	18	20	21	17	23	37	13	14	13	15	8	17	28
**% calcareous**	100	98	98	61	93	37	53	100	99	99	70	68	47	68
**% > 1 mm**						0.3	2.2						0.5	0.9

**Table 5:** Densities of living and dead benthic foraminifera from sediment-surface samples retrieved in 2004 (replication B).

	**Live foraminifera (N)**							**Dead foraminifera (N)**					
Station no.	242	241	240	239	238	237	236	242	241	240	239	238	237
*Adercotryma glomerata*						1.8	27.0						0.6
*Ammodiscus sp.*				0.5		0.6	4.0						0.6
*Ammotium cassis*						9.8	1.0						1.8
*Angulogerina fluens*							13.0						
*Astacolus hyalacrulus*				0.4							0.2		
*Astrononion gallowayi*					0.3		1.0	0.1			0.2		
*Buccella frigida*		0.0	5.0	2.9			2.0	0.0	0.0	0.7			
*Cassidulina obtusa*													0.6
*Cassidulina reniforme*	3.6	2.7	43.3	8.4	9.1	1.2	2.0	3.4	2.9	16.3	6.5	9.7	14.2
*Cibicides lobatulus*							1.0	0.2				0.3	1.2
*Cornuspira foliacea*							0.3						
*Cornuspira sp.*				0.2	0.3								0.6
*Dentalina spp.*	0.1	0.0		0.2					0.1				
*Elphidium bartletti*			1.0	0.5		3.1						0.3	2.5
*Elphidium excavatum*												0.3	
*Elphidium excavatum f. clavata*	2.6	4.5	10.7	6.9	2.3	25.2	24.0	0.6	2.4	2.1	0.9	2.6	30.2
*Elphidium incertum*		0.5	2.2						0.0	0.4			
*Elphidium subarcticum*							1.0						
*Epistominella vitrea*						0.6	5.0					0.3	
*Globobulimina spp.*				0.7	0.3	3.7	1.0				0.2	0.3	1.2
*Haynesina orbiculare*		0.8	0.1		0.3				0.1				
*Hyperammina subnodosa*						1.5	9.0						0.6
*Islandiella islandica*												0.3	
*Islandiella norcrossi f. helenae*		0.6	5.9	9.3	8.0	14.8	13.0	0.1	0.2	0.9	1.3	1.7	7.4
*Islandiella norcrossi f. norcrossi*			0.1	0.5		1.8	3.0						0.6
*Labrospira crassimargo*			1.3	14.4	56.0	65.8	32.0		0.0		3.6	2.6	29.5
*Labrospira jeffreisi*							1.0						
*Lagena spp.*	0.3						4.0	0.0					
*Miliolinella sp. 1*				0.2	0.9		9.0						0.6
*Miliolinella sp. 2*			0.3	1.1					0.0	0.3			
*Miliolinella subrotunda*									0.1				
*Nonionella sp.*													0.6
*Nonionella turgida digitata*						1.2							
*Nonionellina labradorica*	0.0	0.6	1.0	17.5	50.9	7.4	69.0	0.4	0.0	0.7	1.6	2.9	6.2
*Polymorphinidae spp.*					0.3	1.8	1.0				0.4		
*Pyrgo williamsoni*	0.0	0.2	2.2	13.6		0.6	6.0		0.2	0.3	0.7		
*Quinqueloculina seminula*		1.0	0.9	0.2	0.3		5.0		0.0	0.1			
*Quinqueloculina sp.*				0.4	2.9		1.0						
*Quinqueloculina stalkeri*	0.4		0.6		0.3		2.0	0.2	0.2	0.7	0.2	0.9	1.2
*Recurvoides turbinatus*				18.7	1.4	64.6	106.0				3.3	0.9	33.2
*Reophax atlantica*						1.8	6.0						4.9
*Reophax scorpiurus* s.l.				0.2	0.3	4.3	29.0			0.1			1.2
*Robertina arctica*						9.8	11.0						0.6
*Silicosigmoilina groenlandica*		0.5	0.7	0.2	0.3	0.6	9.0		0.1				14.2
*Siphonaperta agglutinata*							2.0						
*Spiroplectammina biformis*			0.1										
*Stainforthia loeblichi*							2.0	0.0			1.3		2.5
*Textularia earlandi*			0.1							0.1			
*Textularia torquata*						0.6							
*Triloculina trihedra*				0.2									1.2
*Trochammina nana*				0.2		1.2	9.0				0.4		
*Trochamminella atlantica*						0.6	4.0						
calcareous indefinite												0.3	
**Benthics/10 cm^3^**	7.1	11.5	75.9	97.3	134.0	224.9	415.3	5.2	6.6	23.0	20.7	23.1	158.2
**Benthics counted**	210	259	512	535	469	373	443	152	148	155	114	81	260
**No. of species**	7	11	17	23	17	24	35	10	15	12	14	14	25
**% calcareous**	100	96	97	65	57	32	43	100	98	99	65	85	45
**% > 1 mm**

**Table 6:** Densities of living and dead benthic foraminifera from sediment-surface samples retrieved in 2005.

	**Live foraminifera (N)**								**Dead foraminifera (N)**							
Station no.	013	012	011	010	009	008	007	006	013	012	011	010	009	008	007	006
*Adercotryma glomerata*							0.4	21.3					0.1		0.4	10.7
*Ammodiscus sp.*					0.1		0.4									
*Ammotium cassis*							0.8	1.3							1.7	2.7
*Angulogerina fluens*								5.3							0.4	1.3
*Astacolus hyalacrulus*					0.3											
*Astrononion gallowayi*				0.6		0.5	0.4	5.3				0.2	0.1	0.5	0.4	9.3
*Buccella frigida*				0.2	0.9	0.5	0.4					0.2	0.6		0.4	4.0
*Cassidulina reniforme*		1.8	0.4	16.1	20.5	11.9	5.8	2.7	0.1	0.2	3.5	8.1	4.7	2.4	1.7	12.0
*Cibicides lobatulus*								2.7								5.3
*Cornuspira foliacea*							0.4									
*Cornuspira sp.*																2.7
*Dentalina spp.*		0.1		0.1		0.5										
*Elphidium bartletti*					0.4	1.0	4.2								0.8	4.0
*Elphidium excavatum f. clavata*	21.2	13.4	13.4	7.1	3.4	4.3	11.7	30.7	1.6	2.1	11.3	2.1	4.1	1.0	12.5	74.7
*Elphidium incertum*			0.1	2.1	0.6	0.5	0.4				0.1	0.5	0.3			1.3
*Elphidium subarcticum*																2.7
*Eponides sp.*								1.3								
*Fissurina spp.*								1.3								1.3
*Globobulimina spp.*					0.1	1.0	1.7	1.3					0.3	1.0		
*Haynesina orbiculare*			0.2					1.3			0.1					1.3
*Hippocrepina indivisa*								1.3								
*Hippocrepinella sp.*	3.4				0.3			4.0								
*Hyperammina subnodosa*							6.9	20.0							1.5	7.2
*Islandiella norcrossi f. helenae*			0.1	2.4	20.5	21.4	23.3	5.3		0.1	0.1	0.3	2.1	2.4	1.7	10.7
*Islandiella norcrossi f. norcrossi*					1.1		5.0	2.7					0.3	0.5	0.8	9.3
*Islandiella norcrossi s.l.*					1.4	0.5	0.8	1.3								
*Labrospira crassimargo*				0.2	55.1	33.8	171.7	16.0				0.1	3.8	8.6	9.6	31.0
*Labrospira jeffreisi*								2.7								
*Lagena spp.*			0.2		0.2											
*Miliolinella sp. 1*		0.3	0.3		0.4	0.5		1.3								
*Miliolinella sp. 2*					7.1								0.2			
*Nonionellina labradorica*				1.7	9.1	168.1	35.4	68.0		0.2	0.2	0.2	1.2	2.9	5.4	62.7
*Polymorphinidae spp.*	0.1			0.2				1.3							0.4	1.3
*Pyrgo williamsoni*			3.5	0.9	8.2	1.0	5.8	12.0			0.4	0.1	0.1		0.4	
*Quinqueloculina seminula*			1.5	0.2	0.1	2.9	1.3	4.0			0.2					
*Quinqueloculina sp.*		0.2		0.4	6.5	1.0	0.8						0.5		0.4	
*Quinqueloculina stalkeri*	0.1			0.4	7.9	4.3	1.3	4.0	0.1		0.8	0.5	4.1	1.4		
*Recurvoides turbinatus*					7.9	2.4	86.7	81.3					0.7		11.3	29.3
*Reophax atlantica*							3.8	1.3	0.3							2.7
*Reophax cf. fusiformis*					2.6			13.3					0.1			37.3
*Reophax scorpiurus* s.l.						1.0	1.7	18.7							16.7	22.8
*Rhabdammina abyssorum*							0.2								0.2	0.3
*Robertina arctica*						0.5	4.6	6.7					0.2			
*Silicosigmoilina groenlandica*			0.1	0.1	0.1		2.5	8.0				0.2			5.0	12.0
*Siphonaperta agglutinata*					0.2		1.3	5.3								4.0
*Stainforthia loeblichi*				0.1	1.0	0.5	0.8	2.7					0.7		0.4	1.3
*Textularia earlandi*																1.3
*Triloculina trihedra*		0.1		0.3	2.5								1.2			
*Trochammina nana*													0.1			5.3
*Trochamminella atlantica*																2.7
**Benthics/10 cm^3^**	24.7	15.8	19.7	33.2	158.5	257.6	380.4	356.0	2.0	2.6	16.5	12.6	25.5	20.5	72.1	374.7
**Benthics counted**	390	275	319	405	1585	541	930	372	32	45	268	154	255	43	177	323
**No. of species**	4	6	10	17	27	21	29	33	4	4	9	11	21	9	21	31
**% calcareous**	86	100	100	99	58	86	28	47	84	100	100	98	81	58	36	56
**% > 1 mm**							1.9	5.6							2.3	2.1

**Table 7:** Densities of living and dead benthic foraminifera from sediment-surface samples retrieved in 2006 (replication A).

	**Live foraminifera (N)**								**Dead foraminifera (N)**							
Station no.	191	178	180	182	184	190	186	188	191	178	180	182	184	190	186	188
*Adercotryma glomerata*	18.5								11.1							
*Ammodiscus sp.*	1.2								3.1	1.5						
*Ammotium sp.*	1.2															
*Angulogerina fluens*	9.2								9.8							
*Armorella sp.*									3.1			0.9	1.3			
*Astacolus hyalacrulus*									0.6							
*Astrononion gallowayi*	2.5	4.6		0.9	1.8				19.1							
*Bolivina pseudopunctata*											1.6					
*Buccella frigida*		1.5	1.6	5.6					1.2	6.1		1.9	0.4			
*Buccella tenerrima*	1.2								14.2							
*Bulimina marginata*									0.6							
*Cassidulina reniforme*	9.8	54.9	222.4	66.8	41.8	1.9	0.5		57.8	15.2	72.0	51.8	51.1	0.1		
*Cibicides lobatulus*	3.1		1.6						11.7							
*Cornuspira sp.*	0.6	1.5	1.6		0.4			0.1								
*Cuneata arctica*									0.6							
*Dentalina spp.*	0.6		0.1		0.9	0.1			1.2							
*Elphidium bartletti*	4.3		3.2						10.5							
*Elphidium excavatum f. clavata*	42.5	131.0	49.6	12.2	93.8	54.7	34.3	9.2	143.4	30.5	14.4	14.1	32.0	4.3	0.9	0.2
*Elphidium frigidum*									1.2							
*Elphidium incertum*	0.6	1.5	1.6		3.1	0.2			0.6				0.4			
*Epistominella vitrea*	3.7			0.9					4.9							
*Fissurina spp*	0.6															
*Globobulimina turgida*			1.6						1.2		1.6	2.8				
*Hippocrepina indivisa*	9.8			0.9					3.1							
*Hyperammina subnodosa*	7.5	2.6							4.4							
*Islandiella islandica*									0.6		1.6					
*Islandiella norcrossi f. helenae*	13.5	32.0	41.6	33.9	1.8				25.2	6.1		5.6	0.4			
*Islandiella norcrossi f. norcrossi*	0.6	1.5		1.9	0.4				1.8							
*Islandiella norcrossi s.l.*									1.2							
*Labrospira crassimargo*	52.1	236.9	89.7	23.5					42.6	24.4	8.0	2.8				
*Lagena spp.*	0.6								1.2							
*Miliolinella sp. 1*	0.6	4.6	1.6			0.1	0.2									
*Miliolinella sp. 2*		1.5		16.0	2.7	0.1						0.9				
*Nonionellina labradorica*	31.4	53.3	145.6	99.8	9.8				97.8	7.6	8.0	4.7	0.4			
*Oolina sp.*									0.6							
*Polymorphinidae spp.*	1.2	1.5	1.6			0.1			4.3							
*Pyrgo williamsoni*	9.8	30.5	6.4	7.5	4.4	0.1			1.2			1.9				
*Quinqueloculina seminula*	1.2	1.5	4.8			0.2										
*Quinqueloculina sp.*		1.5	3.2		1.8						1.6					
*Quinqueloculina stalkeri*	15.4	1.5	11.2	77.2	24.9	0.1		0.1	1.2	1.5	4.8	12.2	3.6			
*Recurvoides turbinatus*	64.0	70.1	6.4	3.8					69.5	9.1		0.9				
*Reophax atlantica*	19.1			0.9					20.9							
*Reophax scorpiurus* s.l.	46.2	30.5	1.6	0.9					77.5	1.5		0.9	0.4			
*Robertina arctica*	16.0	9.1	8.0	13.2	14.2											
*Rosalina sp.*	9.8								0.6							
*Silicosigmoilina groenlandica*	9.8	1.5							14.2	3.0						
*Siphonaperta agglutinata*	17.2	3.0		1.9					4.3			0.9				
*Spiroplectammina biformis*	1.8				0.4				3.7							
*Stainforthia loeblichi*	5.5	3.0		1.9	0.9				14.8							
*Textularia earlandi*	3.1	4.6	9.6	2.8	5.3	0.1			8.6			1.9	0.4			
*Textularia torquata*		1.5							0.6							
*Triloculina trihedra*	0.6			2.8	0.4				0.6			5.6	0.4			
*Trochammina bullata*									0.6							
*Trochammina nana*	1.8								7.4							
*Trochamminella atlantica*	1.8	1.5							2.5							
agglutinated indefinite			1.6		1.3											
**Benthics/10 cm^3^**	440.5	689.0	616.2	375.5	210.2	57.6	35.0	9.3	707.3	106.7	113.6	110.1	91.1	4.4	1.2	0.2
**Benthics counted**	805	484	387	399	473	605	403	167	1201	70	71	117	205	46	14	4
**No. of species**	40	27	23	21	19	11	3	3	46	11	9	16	11	2	1	1
**% calcareous**	46	49	82	91	97	100	100	100	61	63	93	93	98	100	100	100
**% >1 mm**	1.8	0.5	0.03						0.6

**Table 8:** Densities of living and dead benthic foraminifera from sediment-surface samples retrieved in 2006 (replication B).

	**Live foraminifera (N)**							**Dead foraminifera (N)**						
Station no.	191	178	180	182	184	190	188	191	178	180	182	184	190	188
*Adercotryma glomerata*	3.1	1.5						12.9						
*Ammodiscus sp.*		1.5		1.4	0.3							0.3		
*Ammotium sp.*	3.1								4.4					
*Angulogerina fluens*	4.3							4.3						
*Astacolus hyalacrulus*				1.4										
*Astrononion gallowayi*	4.3				0.7			9.2	1.5					
*Buccella frigida*		2.9		19.5	1.0			4.3	2.9					
*Buccella tenerrima*								10.5						
*Cassidulina reniforme*	3.7	61.1	136.3	61.2	92.0	3.4	0.6	27.1	29.1	26.4	48.7	42.0		0.1
*Cassidulina teretis*								0.6						
*Cibicides lobatulus*	4.3		1.4					15.4						
*Cornuspira sp.*	0.6				0.7									
*Dentalina spp.*			1.4					0.6						
*Egerella advena*								0.6						
*Elphidium bartletti*	5.5	1.5			0.7			10.5	1.5		2.8			
*Elphidium excavatum f. clavata*	18.5	62.5	8.3	11.1	95.7	51.6	28.8	80.0	24.7	9.7	11.1	19.7	12.2	1.3
*Elphidium frigidum*								0.6						
*Elphidium incertum*				1.4	1.7			1.2			1.4			
*Elphidium subarcticum*								1.8						
*Epistominella vitrea*	0.6			1.4				1.2						
*Globobulimina turgida*	0.6	2.9	2.8					2.5	2.9	1.4	1.4			
*Hippocrepina indivisa*	1.2			1.4				4.3	1.5					
*Hyperammina subnodosa*	2.0	4.7					0.1	2.3	0.4					
*Islandiella islandica*								1.8				0.3		
*Islandiella norcrossi f. helenae*	3.7	32.0	15.3	51.5	1.3			19.7	1.5	1.4	8.3	1.7		
*Islandiella norcrossi f. norcrossi*	1.2	1.5		1.4				3.1						
*Labrospira crassimargo*	19.9	323.4	108.7	105.8	1.7			32.8	38.5	4.2	2.8			
*Lagena spp.*								0.6						
*Miliolinella sp. 1*		8.7		2.8										
*Miliolinella sp. 2*				13.9	6.3									
*Nonionellina labradorica*	15.4	50.9	107.1	61.2	7.3			73.2	8.7	5.6	2.8	0.0		
*Polymorphinidae spp.*		2.9			0.3			3.1				0.3		
*Pyrgo williamsoni*	4.3	21.8	1.4	33.4	1.0			0.6	2.9					
*Quinqueloculina seminula*						0.8								
*Quinqueloculina sp.*		5.8		1.4	1.3									
*Quinqueloculina stalkeri*	2.5	4.4	9.7	97.4	24.7	0.2	0.1	1.2	2.9	4.2	7.0	7.7		0.1
*Recurvoides turbinatus*	23.4	119.3	4.2	7.0	0.3			47.4	17.5					
*Reophax atlantica*	8.0			1.4	0.0			20.9						
*Reophax scorpiurus* s.l.	23.4	40.7	1.4	1.4	1.7			33.8	11.6					
*Reophax sp.*	0.6							8.6						
*Robertina arctica*	11.1	14.5	5.6	2.8	3.7									
*Rosalina sp.*	6.8													
*Silicosigmoilina groenlandica*	8.6	5.8						18.5	7.3			0.3		
*Siphonaperta agglutinata*	1.8	5.8							1.5					
*Spiroplectammina biformis*		1.5		1.4	2.3			1.8				0.7		
*Stainforthia loeblichi*	2.5	1.5		2.8				11.1	2.9					
*Sygmoilina sp.*				2.8										
*Textularia earlandi*			2.8		8.7			1.2			2.8	0.3		
*Triloculina trihedra*					0.3						4.2	0.3		
*Trochammina nana*								8.6						0.1
*Trochamminella atlantica*	0.6							3.1						
agglutinated indefinite			1.4											
calcareous indefinite		1.5					0.1							
**Benthics/10 cm^3^**	185.6	780.5	407.8	487.0	254.0	56.4	29.7	481.2	163.9	52.9	93.2	73.8	12.2	1.5
**Benthics counted**	327	590	295	351	762	296	564	810	123	38	67	222	64	29
**No. of species**	29	25	15	24	23	4	5	38	19	7	11	12	1	4
**% calcareous**	49	36	71	75	94	100	100	59	51	92	94	98	100	97
**% > 1 mm**	1.20	0.66	0.04	0.02			54.08	0.51	0.61			0.11		72.41

**Table 9:** Densities of living and dead benthic foraminifera from sediment-surface samples retrieved in 2007 (replication A).

	**Live foraminifera (N)**								**Dead foraminifera (N)**							
Station no.	8A+C	7A+C	6A	5A	4A	3A	2A	1A	8A+C	7A+C	6A	5A	4A	3A	2A	1A
*Adercotryma glomerata*								18.0								16.0
*Ammotium cassis*							4.0	24.0								4.0
*Angulogerina fluens*								18.0								4.0
*Armorella sp.*								2.0								4.0
*Astacolus hyalacrulus*							2.0									
*Astrononion gallowayi*								2.0						2.0		24.0
*Buccella frigida*					2.0								4.0	4.0		
*Buccella tenerrima*							2.0	2.0							2.0	8.0
*Cassidulina reniforme*		1.4	19.0	144.0	11.0	140.0	32.0	4.0	0.3	0.4	4.5	162.0	57.5	66.0	48.0	34.0
*Cibicides lobatulus*						2.0		4.0								14.0
*Cornuspira foliacea*						0.4										
*Cornuspira sp.*		0.1					4.0									
*Cuneata arctica*													0.5			
*Dentalina spp.*			0.5	2.0				0.3								2.0
*Elphidium bartletti*			0.5		0.5		12.0						0.5		8.0	12.0
*Elphidium excavatum f. clavata*	5.9	8.3	73.0	284.0	18.5	168.0	44.0	8.0	1.5	14.9	50.5	142.0	16.5	32.0	80.0	96.0
*Elphidium frigidum*																4.0
*Elphidium incertum*				2.0	2.0	2.0						2.0			2.0	
*Elphidium subarcticum*																2.0
*Epistominella vitrea*			0.5		1.0			8.0								4.0
*Fissurina spp.*							2.0									2.0
*Globobulimina spp.*					3.0		2.0						1.0		2.0	4.0
*Hippocrepina indivisa*								34.0								
*Hyperammina subnodosa*							4.8	20.0							0.8	1.5
*Islandiella islandica*															2.0	
*Islandiella norcrossi f. helenae*					6.5	56.0	18.0	12.0					5.5	2.0	10.0	32.0
*Islandiella norcrossi f. norcrossi*													0.5			
*Labrospira crassimargo*					8.5	422.5	186.5	156.5					3.5	14.1	54.1	56.3
*Miliolidae spp.*		0.4														
*Miliolinella sp. 1*							4.0			0.4						
*Miliolinella sp. 2*		4.9	9.0	14.0	3.0	4.0				0.1						
*Nonionella turgida digitata*								2.0								2.0
*Nonionellina labradorica*				30.0	87.5	94.0	120.0	76.0	0.1	0.3		4.0	4.0	6.0	40.0	94.0
*Oolina sp.*																2.0
*Polymorphinidae spp.*		0.1	3.0			2.0	6.0	2.0	0.1							6.0
*Proteonina atlantica*								18.0								2.0
*Pyrgo williamsoni*				12.0	42.5	6.0	4.0	8.0					2.0			
*Quinqueloculina seminula*			1.0			6.0	2.0									2.0
*Quinqueloculina sp.*				4.0	1.5	32.0	10.0						1.0			
*Quinqueloculina stalkeri*	2.1	15.4	31.0	22.0	1.5	14.0	4.0	2.0	0.1	2.0	3.5	30.0	32.5	6.0	2.0	
*Recurvoides turbinatus*					1.0	46.0	62.0	110.0					1.0	2.0	14.0	80.0
*Reophax atlantica*						4.0		20.0								6.0
*Reophax dentaliniformis*												2.0				
*Reophax scorpiurus* s.l.				2.0	0.5	2.1	2.0	76.0						2.0	4.0	26.0
*Reophax sp.*				14.0	8.0	86.0	78.0	38.0				8.0	1.5	28.0	42.0	104.0
*Robertina arctica*				10.0	7.5	26.0	28.0	42.0				4.0	0.5			2.0
*Rosalina sp.*						2.0	2.0	10.0								2.0
*Silicosigmoilina groenlandica*							2.0	6.0					0.5		4.0	8.0
*Siphonaperta agglutinata*					2.0			4.0								
*Spiroplectammina biformis*				4.0												2.0
*Stainforthia loeblichi*							4.0	4.0							2.0	26.0
*Textularia earlandi*				6.0	1.0			6.0				8.0	0.5	8.0		6.0
*Triloculina trihedra*					0.5											
*Trochammina nana*								10.0								4.0
*Trochamminella atlantica*						2.0		4.0								
agglutinated indefinite								2.0								
**Benthics/10 cm^3^**	8.0	30.5	137.5	550.0	209.5	1117.0	641.3	752.8	2.1	18.0	58.5	362.0	133.0	172.1	316.9	697.8
**Benthics counted**	64	244	275	275	419	566	360	532	17	144	117	181	266	87	165	362
**No. of species**	2	7	9	14	21	21	26	34	5	6	3	9	18	12	17	36
**% calcareous**	100	100	100	95	91	50	47	28	100	100	100	95	94	69	62	54
**% > 1 mm**						0.1	0.8	2.8						0.1	0.3	0.3

**Table 10:** Densities of living and dead benthic foraminifera from sediment-surface samples retrieved in 2007 (replication B).

	**Live foraminifera (N)**								**Dead foraminifera (N)**							
Station no.	8B+D	7B+D	6B	5B	4B	3B	2B	1B	8B+D	7B+D	6B	5B	4B	3B	2B	1B
*Adercotryma glomerata*								13.0								6.0
*Ammodiscus sp.*						2.0	2.0									
*Ammotium cassis*								6.0								
*Angulogerina fluens*								21.0								6.0
*Astacolus hyalacrulus*													0.5		1.0	
*Astrononion gallowayi*							1.0			0.1					1.0	4.0
*Buccella frigida*					3.5	2.0							3.0	2.0	5.0	2.0
*Buccella tenerrima*							6.0								1.0	1.0
*Cassidulina reniforme*		1.9	8.5	35.0	19.5	56.0	53.0	2.0	0.3	1.1	3.8	47.0	49.5	56.0	137.0	14.0
*Cibicides lobatulus*							2.0	5.0							3.0	7.0
*Cornuspira foliacea*				0.1		0.3										
*Cornuspira sp.*							6.0	1.0								
*Cuneata arctica*								1.0								
*Dentalina spp.*			0.3			2.0									0.1	
*Elphidium bartletti*					1.0		10.0	2.0								5.0
*Elphidium excavatum f. clavata*	12.3	4.9	76.8	99.0	15.5	56.0	57.0		2.4	10.6	102.5	40.0	15.5	20.0	242.0	44.0
*Elphidium incertum*				3.0			1.0					0.5				1.0
*Elphidium subarcticum*																1.0
*Epistominella vitrea*					0.5			4.0								
*Fissurina spp.*								1.0								
*Globobulimina spp.*					2.5	4.0	6.0	1.0							1.0	1.0
*Haynesina orbiculare*											0.8					
*Hippocrepina indivisa*								9.0								
*Hyperammina subnodosa*							3.6	24.9							0.3	1.5
*Islandiella islandica*			0.3													
*Islandiella norcrossi f. helenae*				1.5	10.5	40.0	21.0	18.0					4.0	12.0	11.0	21.0
*Islandiella norcrossi f. norcrossi*								1.0								2.0
*Labrospira crassimargo*					20.1	198.0	71.5	65.1				0.5	2.5	8.0	24.9	14.4
*Lagena spp.*					0.5											
*Miliolidae spp.*										0.1						
*Miliolinella sp. 1*		0.1			0.5		6.0									
*Miliolinella sp. 2*	0.3	0.5	4.8	1.0	5.5		1.0									
*Nonionellina labradorica*				27.0	64.0	130.0	106.0	16.0	0.1	0.1		2.5	4.5	12.0	14.0	29.0
*Polymorphinidae spp.*			1.0	1.5	0.5			2.0			0.8	0.5		4.0	1.0	2.0
*Proteonina atlantica*								1.0								
*Pyrgo williamsoni*			0.3	2.5	16.5	12.0		4.0			0.3					1.0
*Quinqueloculina seminula*			2.0	3.5		6.0	2.0	5.0			0.3					
*Quinqueloculina sp.*					2.5	4.0	7.0						1.0			
*Quinqueloculina stalkeri*	3.8	14.3	13.5	7.0	2.0	6.0		1.0	0.4	1.0	3.0	6.0	18.0	4.0		
*Recurvoides turbinatus*					1.0	32.0	28.0	73.0					1.0	10.0	10.0	15.0
*Reophax atlantica*								20.0					0.5			5.0
*Reophax scorpiurus* s.l.							7.0	37.0							2.0	11.0
*Reophax sp.*				18.5	3.5	146.0	25.0	31.0				7.5	1.0	64.0	21.0	38.0
*Robertina arctica*				6.5	8.5	14.0	25.0	31.0				0.5				
*Rosalina sp.*					3.0		4.0	2.0								1.0
*Silicosigmoilina groenlandica*							2.0	6.0					0.5		4.0	3.0
*Siphonaperta agglutinata*								6.0								
*Spiroplectammina biformis*					0.5										1.0	
*Stainforthia loeblichi*								2.0			0.3		2.5		4.0	8.0
*Textularia earlandi*				0.5	0.5		1.0					2.0		2.0	3.0	
*Triloculina trihedra*						4.0										
*Trochamminella atlantica*								2.0								
agglutinated indefinite				0.5												
calcareous indefinite			0.3													2.0
**Benthics/10 cm^3^**	16.3	21.6	107.5	207.1	182.1	714.3	454.1	414.0	3.1	13.1	111.5	107.0	104.0	194.0	487.3	245.9
**Benthics counted**	130	173	430	415	365	359	483	589	25	105	446	214	208	97	496	259
**No. of species**	3	5	10	15	22	18	25	32	4	6	8	10	14	11	21	27
**% calcareous**	100	100	100	91	86	47	69	30	100	100	100	91	95	57	86	62
**% > 1 mm**				0.1	0.1	0.0	0.9	6.0							0.3	0.8
3. Downcore distribution of living and dead foraminiferans (size fraction > 0.125 mm) in the upper 10 cm of bottom sediments from 4 stations sampled in 2004 (Table 11, Table 12, Table 13, Table 14).

**Table 11:** Densities of living benthic foraminifera in the upper 10 cm of the sediment retrieved in 2004 (samples 238, 236).

Station no.	**238**										**236**									
Sediment depth interval [cm]	0–1	1–2	2–3	3–4	4–5	5–6	6–7	7–8	8–9	9–10	0–1	1–2	2–3	3–4	4–5	5–6	6–7	7–8	8–9	9–10
*Adercotryma glomerata*											2.4		2.9	5.0	3.2					
*Angulogerina fluens*											2.4	5.8		5.0	6.4	1.3				
*Astrononion gallowayi*											4.7			2.5	6.4	2.7				
*Buccella frigida*							0.3			0.3						2.7	3.0	3.7	2.7	2.9
*Cassidulina reniforme*	5.5	7.9	0.8	1.8	0.6				0.4	0.2		8.7				1.3				
*Cibicides lobatulus*							0.2		0.4		2.4			2.5		1.3	3.0			
*Dentalina* spp.		0.7																		
*Elphidium bartletti*	1.3							0.2			2.4									
*Elphidium excavatum f. clavata*	2.3	0.7				0.2					18.8	2.9	5.7	7.5	9.6	4.0	5.9			
*Fissurina* spp.																	3.0		2.7	
*Hippocrepina indivisa*											4.7									
*Islandiella helenae*	3.8	2.9	0.3			0.2	0.7	0.4	0.8	0.6	7.1					1.3	3.0			
*Islandiella norcrossi*	0.3							0.2	0.2		4.7									
*Labrospira crassimargo*	6.3	8.6	2.5	1.2			0.7	1.3	5.8	0.2	30.6	11.6		2.5	3.2					
*Miliolidae*sp.	0.3																			
*Nonionellina labradorica*	35.0	150.0	15.6			0.2	0.2	0.2	1.0		40.0	87.3	20.0	7.5						
*Polymorphinidae*	0.3	7.1	2.8	5.3	0.6	0.3	0.2		0.2			2.9			3.2					
*Pyrgo williamsoni*											2.4									
*Quinqueloculina stalkeri*	1.0																	3.7		
*Recurvoides turbinatus*	1.0	0.7	0.3	0.6							37.6	2.9	2.9	2.5						2.9
*Reophax scorpiurus, curtus*	0.3										25.9	2.9	5.7					3.7		
*Robertina arctica*											2.4									
*Rosalina* sp.			0.3																	
*Silicosigmoilina groenlandica*	0.3	0.7																		
*Trochammina nana*											2.4			5.0		1.3				
**Benthics/10 cm^3^**	57.3	179.3	22.5	8.8	1.2	0.9	2.2	2.3	8.8	1.2	190.6	125.1	37.1	40.0	32.0	16.0	17.8	11.2	5.3	5.7
**Benthics counted**	229	1004	81	30	8	5	13	12	44	8	1296	688	208	256	160	96	96	48	32	32
**No. of species**	13	9	7	4	2	4	6	5	7	4	16	8	5	9	6	8	5	3	2	2
**% calcareous**	86	94	88	80	100	100	69	42	34	88	46	86	69	63	80	92	100	67	100	50

**Table 12:** Densities of living benthic foraminifera in the upper 10 cm of the sediment retrieved in 2004 (samples 242, 239).

Station no.	**242**										**239**									
Sediment depth interval [cm]	0–1	1–2	2–3	3–4	4–5	5–6	6–7	7–8	8–9	9–10	0–1	1–2	2–3	3–4	4–5	5–6	6–7	7–8	8–9	9–10
*Adercotryma glomerata*																0.2				
*Ammodiscus* sp.											0.2	0.4	0.2							
*Angulogerina fluens*																				
*Astrononion gallowayi*																			0.6	
*Buccella frigida*											1.7	0.4		0.2	0.3	0.3				
*Cassidulina reniforme*	4.5	1.6	0.6								4.7	1.5	0.5	0.9		0.2	0.3	0.3		0.2
*Cibicides lobatulus*															0.2			0.3		
*Cornuspira* sp.												0.2								
*Dentalina* spp.	0.3														0.2					
*Elphidium bartletti*												0.4	0.2							
*Elphidium excavatum f. clavata*	0.5	0.3				0.2					1.8	0.6	0.3	0.5	0.8	0.2				
*Islandiella helenae*											5.2	3.5	0.6	0.7	0.6	0.2	0.3			0.2
*Islandiella norcrossi*											1.3	0.4	0.2	0.5						
*Labrospira crassimargo*											8.2	3.7	1.9	0.4	0.3	0.7	0.7	1.0		
*Lagena* spp.	0.2																			
*Miliolidae*sp.	0.5										0.3									
*Miliolinella* sp. 1											0.2									
*Miliolinella* sp. 2												0.2	0.2							
*Nonionellina labradorica*											4.3	35.0	13.1	0.4	0.6					
*Polymorphinidae*	0.2										0.2	2.3	1.5	1.3	0.9	0.2		0.3		
*Pyrgo williamsoni*											2.8	2.3			0.2					
*Quinqueloculina stalkeri*	0.5																			
*Recurvoides turbinatus*											1.5	0.2			0.2					
*Reophax scorpiurus, curtus*																0.2				
*Silicosigmoilina groenlandica*															0.3					
*Stainforthia loeblichi*											0.3	0.8								
*Trochammina nana*											0.7	0.2								
**Benthics/10cm^3^**	6.6	1.9	0.6	0.0	0.0	0.2	0.0	0.0	0.0	0.0	33.3	51.9	18.5	4.8	4.5	2.1	1.3	2.0	0.6	0.4
**Benthics counted**	42	13	3	0	0	1	0	0	0	0	200	270	115	27	29	12	8	12	4	2
**No. of species**	7	2	1	0	0	1	0	0	0	0	15	16	10	8	11	8	3	4	1	2
**% calcareous**	100	100	100	0	0	100	0	0	0	0	69	91	89	93	83	50	50	50	100	100

**Table 13:** Densities of dead benthic foraminifera in the upper 10 cm of the sediment retrieved in 2004 (samples 238, 236).

Station no.	**238**										**236**									
Sediment depth interval [cm]	0–1	1–2	2–3	3–4	4–5	5–6	6–7	7–8	8–9	9–10	0–1	1–2	2–3	3–4	4–5	5–6	6–7	7–8	8–9	9–10
*Adercotryma glomerata*											9.4	17.5	20.0	55.0	41.6	16.0	29.6		29.3	14.3
*Ammodiscus* sp.											4.7			12.5	9.6	2.7	3.0		5.3	20.0
*Angulogerina fluens*								0.4			4.7	17.5	2.9	15.0	16.0	10.7	8.9	3.7	8.0	8.6
*Astrononion gallowayi*												20.4	17.1	15.0	16.0	8.0	11.9	22.3		34.3
*Buccella frigida*						0.2	0.5	0.6	1.8	1.2	16.5	14.5	8.6	25.0	28.8	17.3	32.6	40.9	29.3	28.6
*Cassidulina reniforme*	1.0	3.6	4.2	15.6	5.7	7.9	11.6	22.3	23.4	17.3	49.4	81.5	237.1	127.5	112.0	56.0	88.9	67.0	112.0	145.7
*Cibicides lobatulus*		0.7				0.2					7.1	14.5	20.0	20.0	9.6	5.3	32.6	11.2	24.0	22.9
*Cornuspira* sp.							0.2		0.4	2.4				2.5			5.9	3.7	2.7	
*Dentalina* spp.																	3.0		2.7	
*Elphidium bartletti*	0.5		0.3	3.5			0.2	0.2	0.4	1.1	18.8	8.7	8.6	12.5	32.0	28.0	59.3	44.7	34.7	54.3
*Elphidium excavatum f. clavata*	0.5	1.4	3.1	13.8	6.5	4.8	6.0	6.9	4.6	2.6	115.3	177.5	114.3	297.5	297.6	114.7	225.2	249.3	250.7	302.9
*Fissurina* spp.															3.2	2.7	5.9			2.9
*Hippocrepina indivisa*											4.7	2.9							2.7	
*Islandiella helenae*	0.8	5.0	1.1	3.5	2.4	0.7	2.8	0.8	2.8	2.3	18.8	29.1	20.0	22.5	44.8	13.3	47.4	33.5	13.3	54.3
*Islandiella norcrossi*			0.3				0.2	0.2		0.2		2.9	22.9	12.5	12.8	9.3	23.7	7.4	13.3	2.9
*Labrospira crassimargo*	0.5	3.6	3.1	5.6	2.5	4.1	8.3	6.2	8.4	7.9	40.0	64.0	62.9	90.0	115.2	32.0	154.1	85.6	128.0	108.6
*Lagena* spp.											7.1	8.7			6.4	2.7			2.7	
*Nonionellina labradorica*	1.0	10.7	2.2	10.3	3.7	2.4		4.6	6.2	6.8	94.1	212.4	185.7	160.0	176.0	58.7	189.6	171.2	144.0	197.1
*Polymorphinidae*					1.6	0.5	0.3	0.4		0.2	7.1	8.7	11.4		9.6	4.0	14.8		8.0	11.4
*Pyrgo williamsoni*								0.2		0.3										
*Quinqueloculina stalkeri*		2.1	0.6	5.0	1.0	0.3	0.3	1.9	1.2	1.2	4.7	2.9		7.5	12.8	1.3	3.0		2.7	2.9
*Recurvoides turbinatus*			2.2			1.0	0.2	1.2	0.2	1.8	21.2	26.2	40.0	30.0	44.8	8.0	35.6	14.9	26.7	31.4
*Reophax scorpiurus, curtus*			0.3			0.7	0.9	1.2	0.4	0.8	75.3	130.9	54.3	55.0	54.4	26.7	53.3	44.7	58.7	51.4
*Robertina arctica*											2.4									
*Rosalina* sp.		0.7																		
*Silicosigmoilina groenlandica*					0.6		0.3	0.4	0.4	0.6	14.1	26.2	14.3	20.0	12.8					
*Spiroplectammina biformis*							0.3		0.2	0.2	2.4	2.9	11.4	25.0	19.2	1.3	14.8	3.7		2.9
*Stainforthia loeblichi*							0.2	0.4	0.6	1.1	25.9	8.7	5.7	15.0	28.8	13.3	20.7	22.3	26.7	28.6
*Triloculina trihedra*								0.2		0.3							3.0			8.6
*Trochammina nana*											9.4	2.9	28.6	17.5	35.2	2.7	11.9	29.8	37.3	34.3
*Trochamminella atlantica*											2.4	2.9		2.5	6.4	2.7		3.7		
**Benthics/10 cm^3^**	4.3	27.9	17.2	57.4	24.0	22.9	32.2	47.9	51.0	48.0	555.3	884.4	885.7	1040.0	1145.6	437.3	1078.5	859.5	962.7	1168.6
**Benthics counted**	17	156	62	195	163	133	187	249	255	317	3776	4864	4960	6656	5728	2624	5824	3696	5776	6544
**No. of species**	6	8	10	7	8	11	15	17	14	18	23	23	19	22	24	23	24	18	22	22
**% calcareous**	88	87	68	90	87	74	68	82	80	72	67	69	74	70	70	79	71	78	70	78

**Table 14:** Densities of dead benthic foraminifera in the upper 10 cm of the sediment retrieved in 2004 (samples 242, 239).

Station no.	**242**										**239**									
Sediment depth interval [cm]	0–1	1–2	2–3	3–4	4–5	5–6	6–7	7–8	8–9	9–10	0–1	1–2	2–3	3–4	4–5	5–6	6–7	7–8	8–9	9–10
*Adercotryma glomerata*												0.2	0.2			0.2		0.3	0.6	
*Ammodiscus* sp.												1.0	1.0	1.3	1.6	0.3	2.3	4.7	4.0	1.6
*Angulogerina fluens*																				0.2
*Astrononion gallowayi*																	0.3	0.3		0.4
*Buccella frigida*		0.3					0.2				0.3	1.0	4.0	3.9	5.3	4.3	8.7	10.0	1.7	1.1
*Cassidulina reniforme*	3.0	8.8	4.6	1.7	10.7	12.1	7.6	18.1	14.6	5.0	2.0	6.3	4.8	6.6	14.7	31.2	92.3	114.3	48.0	33.0
*Cibicides lobatulus*		0.3		0.2					0.2	0.2				0.2			0.3	0.3		
*Cornuspira* sp.																0.2		0.7		
*Dentalina* spp.	0.2	0.1		0.2	0.2														0.6	
*Elphidium bartletti*	0.2	1.0									0.2	0.2		0.4				1.3	0.6	
*Elphidium excavatum f. clavata*	0.3	0.7	1.2	0.7	0.4	1.4	1.6	8.5	12.2	9.4	0.7	4.4	18.9	24.6	12.2	5.0	25.3	66.7	16.0	6.8
*Fissurina* spp.																			0.6	
*I. helenae*			0.2								1.3	2.7	3.4	0.9	2.7	2.2	5.0	11.3	5.7	4.5
*I. norcrossi*											0.3	0.4	0.8	0.4	0.9	0.7	2.3	2.0	2.9	0.4
*Labrospira crassimargo*											1.7	0.8	4.0	3.9	4.4	4.3	6.0	13.0	3.4	6.3
*Lagena* spp.						0.2		1.7				0.2				0.3	0.3	1.3	1.1	0.4
*Nonionellina labradorica*	0.2	1.5	0.6	0.2	0.2			0.2			0.8	3.8	2.1	0.7	2.0	2.2	3.0	3.0	1.1	1.6
*Polymorphinidae*		0.3								0.2		0.2		0.4	0.3	0.2	2.3	2.3		0.5
*Pyrgo williamsoni*		0.3											0.2							0.2
*Quinqueloculina stalkeri*	0.8	1.6	0.2			0.2	0.6	11.7	15.0	8.5						0.3		2.0	0.6	0.7
*Recurvoides turbinatus*													0.6	0.2	0.9	0.7	0.3	1.0	1.1	
*Reophax scorpiurus, curtus*																0.2	1.3	5.0	4.0	0.4
*Rosalina* sp.																0.3	0.3	0.3	0.6	0.2
*Silicosigmoilina groenlandica*	0.3																	1.3	6.9	
*Spiroplectammina biformis*					0.2							0.4								
*Stainforthia loeblichi*											0.3			0.5	0.9	1.2	0.7	1.0		0.7
*Triloculina trihedra*												0.2				0.2	0.3	0.3	1.1	0.2
*Trochammina nana*												0.4				0.2	0.3	1.0		
**Benthics/10 cm3**	4.8	15.0	6.8	3.0	11.7	13.8	10.0	40.2	42.0	23.2	7.7	22.1	40.0	43.9	45.9	54.3	151.7	243.7	100.6	58.9
**Benthics counted**	31	102	34	14	63	80	62	193	210	153	46	115	248	246	294	315	910	1462	704	330
**No. of species**	7	10	5	5	5	4	4	5	4	5	9	15	11	13	11	19	18	23	19	18
**% calcareous**	94	100	100	100	98	100	100	100	100	100	78	88	85	88	85	89	93	89	80	86

## 2 Experimental design, materials and methods

### 2.1 Sampling and laboratory procedure

**Fig. 1:**
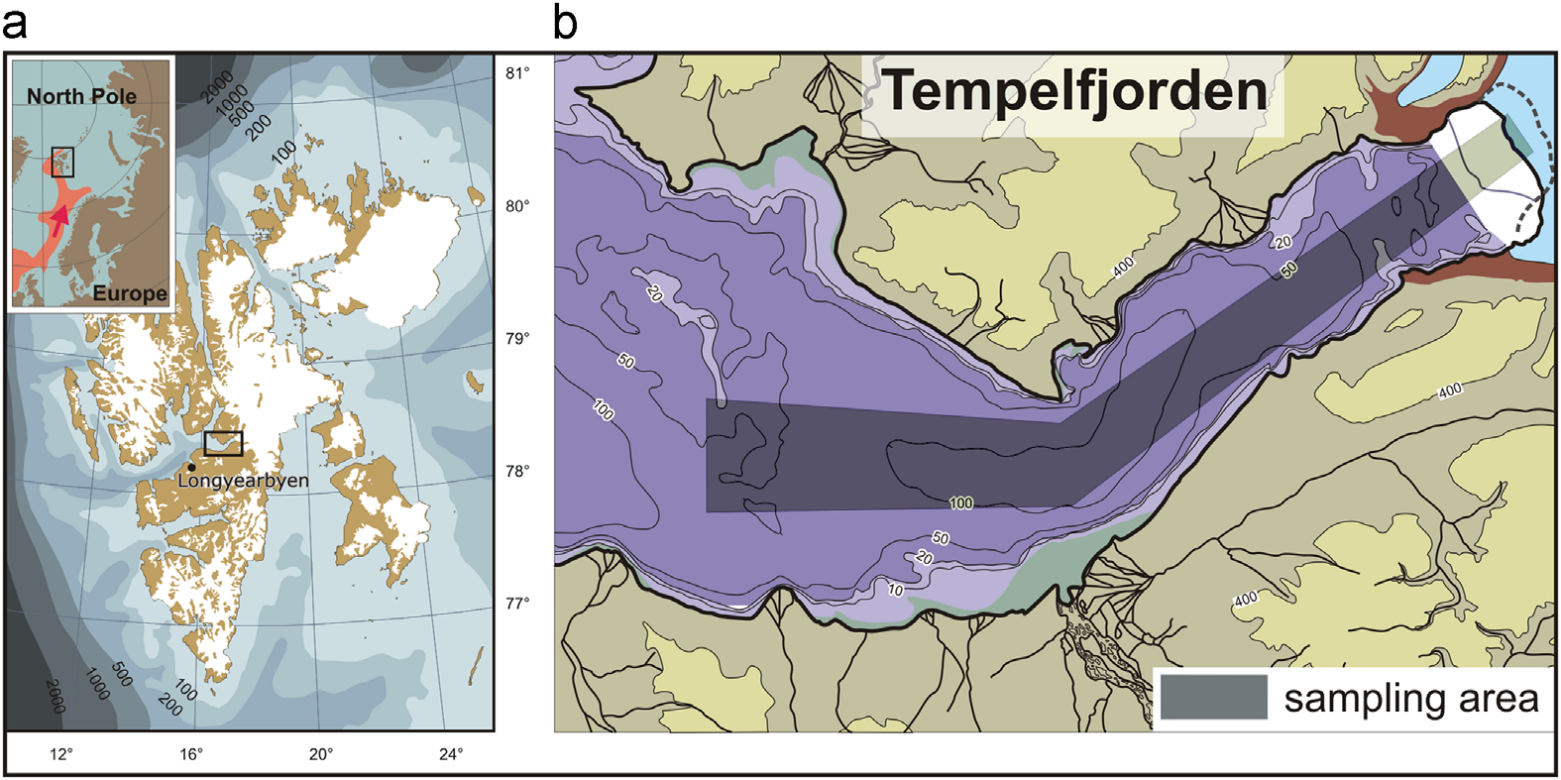
The sampling area. A. Svalbard. B. Tempelfjorden.

Samples were obtained during 8 summer cruises of research vessels to Tempelfjorden in 1995 and 2001–2007 (Fig. 1, Table 1). Sediment samples were retrieved using a box corer or interface corer. A volume of 80 to 200 cm^3^ of seafloor sediments from the 0–2-cm interval was sampled at each station. Additionally, in 2004, four short cores were obtained to reveal the vertical distribution of dominant and common species at different distances from the glaciers in the fjord head. Samples were preserved with 96% alcohol solution of Rose Bengal dye (1 g/L) in order to distinguish living specimens from dead. In the laboratory, samples were washed on sieves with the 0.125 mm and 1 mm mesh sizes and dried in an oven at 80 °C. Dry samples were split using a dry splitter. From each split a minimum of 300 living and dead foraminifera were identified to the lowest possible taxon and counted under a dissecting microscope. Table 2, Table 3, Table 4, Table 5, Table 6, Table 7, Table 8, Table 9, Table 10, Table 11, Table 12, Table 13, Table 14 show pulled data for both size fractions (0.125 mm and 1 mm) with the proportion of larger fraction provided at the bottom of each table.
